# Association of Three Polymorphisms rs11614913, rs2910146, and rs3746444 in miRNA-196a2, miRNA-146a, and miRNA-499 with Inflammatory Bowel Disease: A Systematic Review and Meta-Analysis

**DOI:** 10.1155/2018/7295131

**Published:** 2018-03-07

**Authors:** Ying Liu, Lingxin Xiong, Yan Zhou, Bingzhen Zheng, Tongjun Liu, Wei Xie

**Affiliations:** ^1^Department of General Surgery, The Second Hospital of Jilin University, Changchun, Jilin, China; ^2^School of Pharmaceutical Sciences, Jilin University, Changchun, China; ^3^National & Local Joint Engineering Research Center for Ginseng Innovative Drugs Development, Changchun, China; ^4^School of Business Administration, South China University of Technology, Guangzhou, China

## Abstract

**Background:**

It has been found that single-nucleotide polymorphisms (SNPs) of microRNA might be involved in the development of inflammatory bowel diseases (IBDs). However, the related retrospective research has not been reported. In this work, we performed a meta-analysis to derive a more precise estimation of the associated relationship.

**Methods:**

We searched the studies on the association of SNPs of microRNA with the hereditary susceptibility of IBD in PubMed and Embase; eligible research was selected by screening the abstract and full text. The meta-analysis was performed based on the statistical software Stata 14.0, and besides, the odds ratio and 95% confidence interval were calculated to evaluate the strength of the association.

**Results:**

159 papers were acquired from the PubMed and Embase databases, and five eligible articles containing nine case-control studies were selected. In the study, we first found that the association between *miRNA-196a2 rs11614913* and IBD was insignificant. Then, the susceptibility of *miRNA-146a rs2910146* to IBD increased significantly in allelic comparison, homozygote model, heterozygote model, and dominant model. Moreover, a positive relationship between *miRNA-499 rs3746444* and IBD was identified in the homozygote model.

**Conclusion:**

Our findings demonstrated that *miRNA-146a rs2910146* (G>C) polymorphism was associated with the susceptibility to IBD and *miRNA-196a2 rs11614913* (T>C) and *miRNA-499 rs3746444* (A>G) did not reveal an obvious relationship with the IBD susceptibility.

## 1. Introduction

Inflammatory bowel diseases (IBDs) are chronic multifactorial and relapsing inflammatory disorders of the gastrointestinal tract, including Crohn's disease (CD) and ulcerative colitis (UC). In recent years, the population of patients suffering the IBDs shows an increasing trend. The complex interaction among host immune system, intestinal flora, and environmental factors is considered to be the cause of the IBD pathogenesis [1, 2]. Hence, the identification of genetic factors involved in the development of IBD has attracted attention in the current research area [3].

MicroRNAs (miRNAs) are an abundant class of small, noncoding, and single-stranded RNAs, which play an important role in many biological processes, such as cellular proliferation, differentiation, apoptosis, immune response, and signal transduction by regulating gene expression [4]. It has been reported that many diseases, for example, inflammatory diseases and various cancers, will implicate an miRNA dysregulation [5, 6]. Though the majority of studies are focused on the miRNA expression, a considerable number of publications have reported that the single-nucleotide polymorphisms (SNPs) in miRNA genes could be linked to the genetic susceptibility to disease development.

In the literature, the scholars have reported several miRNA-SNPs, including *miRNA-196a2 rs11614913*, *miRNA-146a rs2910146*, and *miRNA-499 rs3746444*, that are associated with the susceptibility to various diseases such as rheumatoid arthritis (RA) [7, 8], systemic lupus erythematosus (SLE), and various cancers [9, 10]. In addition, some research has been proposed to investigate the association between the SNPs and the risk of IBD [11–15], while the results were inconclusive or even conflicting. Therefore, we performed a meta-analysis to examine how *miRNA-196a2 rs11614913*, *miRNA-146a rs2910146*, and *miRNA-499 rs3746444* polymorphisms will affect the IBD susceptibility.

## 2. Methods and Materials

In this work, the meta-analysis was performed according to the statement guidelines of Preferred Reporting Items for Systematic Reviews and Meta-Analysis (PRISMA) [16] and the Cochrane handbook was used to lead the analysis.

### 2.1. Identification of Eligible Studies

The last search was updated on May 1, 2017. Specifically, we carried out a systematic search in the PubMed and Embase databases with two independent investigators. In the search, the following terms: “microRNA OR mirRNA,” “polymorphism,” and “inflammatory bowel disease OR ulcerative colitis OR Crohn disease,” were adopted and the publication date was not restricted. The reference lists were searched manually to identify potential candidates. Both of the investigators had received professional training in literature review and search, statistics, and evidence-based medicine.

### 2.2. Inclusion and Exclusion Criteria

In this meta-analysis, we also set up inclusion and exclusion criteria to select useful and/or meaningful literature. The selected research should meet the inclusion criteria as follows: (1) evaluation of the association between miRNA196a2/146a/499 and IBD, (2) independent case-control study for human beings, (3) detailed genotype data could be acquired to calculate the odds ratios (ORs) and 95% confidence intervals (CIs), and (4) if multiple studies from the same population and/or the same group were found, we only include the largest study. On the other hand, we apply the following exclusion criteria to eliminate redundant articles: (1) duplication of previous publications, (2) comments, reviews, and editorials, (3) family-based studies of pedigree, and (4) studies without detailed genotype data. In particular, based on the proposed inclusion and exclusion criteria, two investigators (Ying Liu and Lingxin Xiong) were assigned to select the literature independently by screening the title, abstract, and full text, and any dispute was solved by discussion.

### 2.3. Data Extraction

The data of the eligible studies were extracted in duplicate by two investigators independently. In each study, we collected the following information: name of the first author, year of publication, country of origin, ethnicity, characteristics of cases and controls, detective sample, genotyping methods, criteria of IBD, Hardy-Weinberg equilibrium (HWE), number of cases and controls, and genotype frequencies of cases and controls for *miRNA-196a2*, *miRNA-146a*, and *miRNA-499*, respectively. Different ethnic descents were classified as Caucasian and Asian. The two investigators had checked the statistical difference of the extracted data, and they should check up on work until consensus was achieved on every item.

### 2.4. Quality Score Assessment

According to the methodological quality assessment scale modified from previous research (Table 1 [17]), the quality of each selected work was scored by two authors, respectively. Five items were carefully checked based on this scale, including representativeness of cases, source of control, sample size, quality control of genotyping methods, and Hardy-Weinberg equilibrium. We used traditional epidemiological considerations to evaluate the quality scores (range from 0 to 10; a high score indicates the study has a good quality). Again, the two investigators solved the disagreements via discussion.

### 2.5. Statistics Analysis

We followed the guidelines of the PRISMA checklists to conduct the associated meta-analysis. First, the HWE was assessed for each study by the chi-square test in the control group. The odds ratio (OR) and 95% confidence interval (CI) were calculated to evaluate the strength of the association between miRNA196a2/146a/499 SNPs and susceptibility of UC and CD. The pooled ORs were performed for allelic comparison (*miR196a2*: T versus C, *miR146a*: C versus G, and *miR499*: G versus A), homozygote model (*miR196a2*: TT versus CC, *miR146a*: CC versus GG, and *miR499*: GG versus AA), heterozygote model (*miR196a2*: TC versus CC, *miR146a*: CG versus GG, and *miR499*: GA versus AA), dominant model (*miR196a2*: TT + TC versus CC, *miR146a*: CC + CG versus GG, and *miR499*: GG + GA versus AA), and recessive model (*miR196a2*: TT versus TC + CC, *miR146a*: CC versus CG + GG, and *miR499*: GG versus GA + AA). The statistical significance level of the pooled OR was determined by a *Z*-test with a *P* value less than 0.05. Heterogeneity was assessed by the Q statistic (significance level of *P* < 0.1) and I2 statistic (the evidence of significant inconsistency can be revealed when it is greater than 50) [18]. If the heterogeneity was not significant, the fixed effects model was utilized to evaluate the summary OR and 95% CI. Otherwise, the random effects model was implemented.

As a further step, by deleting one study in each turn, the corresponding sensitivity analysis was carried out to evaluate the effect of each study on the combined ORs. Besides, subgroup analyses were stratified based on the ethnicity. All statistical analyses were performed with the software Stata 14.0 (StataCorp, College Station, TX, USA). Note that, except specified conditions, we considered the significance by *P* < 0.05.

## 3. Results

### 3.1. Characteristics of Studies

**Figure 1:**
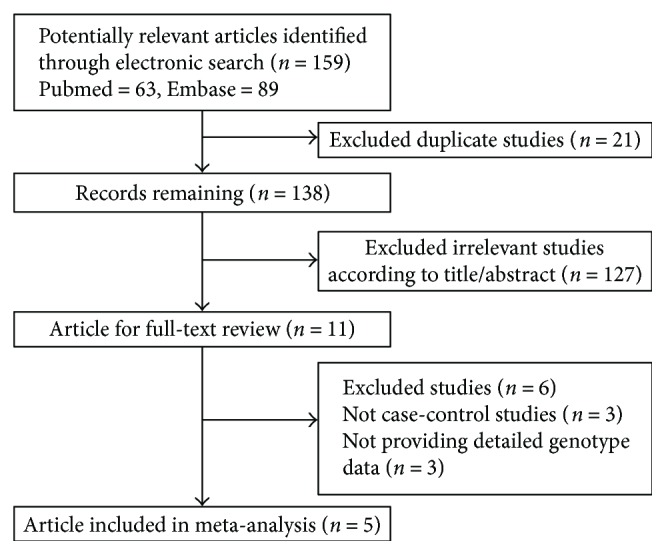
Characteristics of studies included in the meta-analysis.

In this work, we acquired 159 studies in total from the PubMed and Embase databases and the literature review process was summarized as a flow chart shown in Figure 1. In the current study, according to the inclusion and exclusion criteria, 5 eligible case-control studies containing 1761 cases and 1892 controls were chosen in our meta-analysis [11–15]. The characteristics of each study are given in Table 1. Three of them are for Caucasian and the other two are for the Asian population. In each case, the genotype frequencies of *miRNA-196a2 rs11614913*, *miRNA-146a rs2910146* and *miRNA-499 rs3746444* were presented and each of them was treated as a separated study. Different genotyping methods were applied to the studies, including polymerase chain reaction-restriction fragment length polymorphism (PCR-RFLP) [11, 13–15] and allelic discrimination assay by TaqMan® technology [12]. The genotyping distribution was in agreement with the HWE in all studies except the one proposed by Ranjha et al. [11].

### 3.2. Association between miRNA-196a2 and IBD

The pooled ORs among three SNPs and UC are presented in Table 2. We first analyzed the association between the *miRNA-196a2* polymorphism and the IBD susceptibility. When the *Q*-test of heterogeneity was not significant, the fixed effects model was used to conduct analyses. Otherwise, the random effects model was adopted.

Overall, significant statistical heterogeneity was not identified in the homozygote model and recessive model. Therefore, the fixed effects model was implemented and the random effects model was applied to the other three models. In addition, no significant association was identified in all models (T versus C: OR = 0.98, 95% CI 0.85–1.13, *P*_H_ = 0.019; TT versus CC: OR = 0.92, 95% CI 0.76–1.11, *P*_H_ = 0.18; TC versus CC: OR = 1.03, 95% CI 0.74–1.43, *P*_H_< 0.001; TT + TC versus CC: OR = 1.04, 95% CI 0.79–1.36, *P*_H_< 0.001; and TT versus TC + CC OR = 0.97, 95% CI 0.83–1.14, *P*_H_ = 0.12).

Next, we conducted subgroup analyses for ethnicity and diseases. In Caucasian subjects, significant statistical heterogeneity was identified in all models and the random effects model was used. However, the reduction of IBD risk was not significant in each model (T versus C: OR = 0.99, 95% CI 0.78–1.25, *P*_H_ = 0.005; TT versus CC: OR = 0.88, 95% CI 0.60–1.27, *P*_H_ = 0.099; TC versus CC: OR = 1.10, 95% CI 0.66–1.83, *P*_H_< 0.001; TT + TC versus CC: OR = 1.10, 95% CI 0.72–1.66, *P*_H_< 0.001; and TT versus TC + CC: OR = 0.88, 95% CI 0.59–1.30, *P*_H_ = 0.049). On the other hand, in the Asian population, no significant statistical heterogeneity was identified in the models and the fixed effects model was used. In each model, the association was not significant (T versus C: OR = 0.97, 95% CI 0.90–1.07, *P*_H_ = 0.4; TT versus CC: OR = 0.96, 95% CI 0.73–1.25, *P*_H_ = 0.32; TC versus CC: OR = 0.91, 95% CI 0.71–1.16, *P*_H_ = 0.58; TT + TC versus CC: OR = 0.92, 95% CI 0.74–1.16, *P*_H_ = 0.40; and TT versus TC + CC: OR = 1.03, 95% CI 0.83–1.14, *P*_H_ = 0.80).

In the UC, significant statistical heterogeneity was observed in all models, while no significant statistical heterogeneity was found in the CD. Association could not be significantly identified in both subgroups (UC: T versus C: OR = 0.94, 95% CI 0.76–1.17, *P*_H_ = 0.007; TT versus CC: OR 0.79, 95% CI 0.62–1.00, *P*_H_ = 0.23; TC versus CC: OR = 1.07, 95% CI 0.62–1.84, *P*_H_< 0.001; TT + TC versus CC: OR = 1.00, 95% CI 0.64–1.53, *P*_H_< 0.001; TT versus TC + CC OR = 0.87, 95% CI 0.71–1.06, *P*_H_ = 0.11;CD: T versus C: OR = 1.06, 95% CI 0.92–1.22, *P*_H_ = 0.65; TT versus CC: OR = 1.16, 95% CI 0.86–1.56, *P*_H_ = 0.65; TC versus CC: OR = 0.97, 95% CI 0.77–1.22, *P*_H_ = 0.96; TT + TC versus CC: OR1.14, 95% CI 0.92–1.41, *P*_H_ = 0.25; and TT versus TC + CC: OR = 1.16, 95% CI 0.9–1.48, *P*_H_ = 0.57).

### 3.3. Association between miRNA-146a and IBD

The analyses for the association between *miRNA-146a* polymorphism and the risk of IBD were realized by 7 independent studies (studies related to both CD and UC can be seen as two studies). Overall, the random effects model was used in all models due to the significant heterogeneity. The risk of IBD was significantly increased and could be observed in all models (C versus G: OR = 1.34, 95% CI 1.10–1.62, *P*_H_ = 0.001; CC versus GG: OR = 1.56, 95% CI 1.10–2.22; CG versus GG: OR = 1.50, 95% CI 1.17–1.93, *P*_H_ = 0.081; and CC + CG versus GG: OR = 1.54, 95% CI 1.17–2.02, *P*_H_ = 0.022), except the recessive model (CC versus CG + GG: OR = 1.19, 95% CI 0.95–1.49, *P*_H_ = 0.062).  Figure 1 depicts a trend of the increased risk.

Then, the subgroup analyses were concluded. In the Caucasian population, significant heterogeneity was found in all models. Except for the heterozygote model (CG versus GG: OR = 1.50, 95% CI 1.00–2.24, *P*_H_ = 0.051), no significant association was observed in other models (C versus G: OR = 1.39, 95% CI 0.94–2.04, *P*_H_< 0.001; CC versus GG: OR = 1.45, 95% CI 0.68–3.08, *P*_H_ = 0.009; CC + CG versus GG: OR = 1.46, 95% CI 0.90–2.34, *P*_H_ = 0.008; and CC versus CG + GG: OR = 1.18, 95% CI 0.72–1.92, *P*_H_ = 0.019), while the trend of increased risk still existed. In the Asian population, heterogeneity was not significant in each model and an increased susceptibility was observed in all models (C versus G: OR = 1.29, 95% CI 1.12–1.48, *P*_H_ = 0.79; CC versus GG: OR = 1.69, 95% CI 1.27–2.24, *P*_H_ = 0.52; CG versus GG: OR = 1.49, 95% CI 1.31–1.87, *P*_H_ = 0.198; CC + CG versus GG: OR = 1.61, 95% CI 1.24–2.08, *P*_H_ = 0.25; and CC versus CG + GG: OR = 1.27, 95% CI 1.04–1.54, *P*_H_ = 0.886).

In the UC subjects, statistical heterogeneity was not significant in all models. A significant increased risk of UC could be observed in all models (C versus G: OR = 1.32, 95% CI 1.15–1.50, *P*_H_ = 0.64; CC versus GG: OR = 1.43, 95% CI 1.06–1.93, *P*_H_ = 0.39; CG versus GG: OR = 1.32, 95% CI 1.04–1.67, *P*_H_ = 0.42; and CC + CG versus GG: OR = 1.38, 95% CI 1.10–1.73, *P*_H_ = 0.34), except the recessive model (CC versus CG + GG: OR = 1.20, 95% CI 0.99–1.47, *P*_H_ = 0.65). However, the increasing trend of susceptibility was apparent. In the CD subjects, except for the heterozygote model, significant statistical heterogeneity existed. Although the risk reduction still existed in other models (C versus G: OR = 1.37, 95% CI 0.85–2.22, *P*_H_ < 0.001; CC versus GG: OR = 1.94, 95% CI 0.89–4.24, *P*_H_ = 0.009; and CC versus CG + GG: OR = 1.33, 95% CI 0.76–2.33, *P*_H_ = 0.006), an increased risk of CD was found in the heterozygote model (CG versus GG: OR = 1.94, 95% CI 1.49–2.52, *P*_H_ = 0.15) and dominant model (CC + CG versus GG: OR = 1.79, 95% CI 1.10–2.02, *P*_H_ = 0.034).

### 3.4. Association between miRNA-499 and UC

Six independent studies in three articles were included in the assessment of the association between the *miRNA-499* polymorphism and the risk of IBD. In a nutshell, significant statistical heterogeneity was identified in the heterozygote model, dominant model, and recessive model, for which the random effects model was used in the three models. A significant increased risk was identified in the homozygote model (GG versus AA: OR = 1.43, 95% CI 1.06–1.93, *P*_H_ = 0.27), while no obvious association was observed in other models (G versus A: OR = 1.08, 95% CI 0.96–1.22, *P*_H_ = 0.75; GA versus AA: OR = 0.86, 95% CI 0.56–1.33, *P*_H_< 0.001; GG + GA versus AA: OR = 0.97, 95% CI 0.78–1.20, *P*_H_ = 0.064; and GG versus GA + AA: OR = 1.28, 95% CI 0.64–2.53, *P*_H_ = 0.001).

In the Caucasian population, the random effects model was adopted in the heterozygote model and recessive model (due to the significant statistical heterogeneity), and the fixed effects model was implemented in other models. A significant increased risk in the homozygote model (GG versus AA: OR = 1.72, 95% CI 1.19–1.49, *P*_H_ = 0.24) was observed, while no significant association was found in other models (G versus A: OR = 1.08, 95% CI 0.92–1.28, *P*_H_ = 0.38; GA versus AA: OR = 0.62, 95% CI 0.29–1.35, *P*_H_< 0.001; GG + GA versus AA: OR = 0.81, 95% CI 0.66–1.00, *P*_H_ = 0.17; and GG versus GA + AA: OR = 1.74, 95% CI 0.68–4.49, *P*_H_ = 0.016). In the Asian population, no significant statistical heterogeneity was identified in all models and therefore the fixed effects model was used. However, no significant association was found (G versus A: OR = 1.09, 95% CI 0.91–1.30, *P*_H_ = 0.70; GG versus AA: OR = 0.94, 95% CI 0.54–1.64, *P*_H_ = 0.83; GA versus AA: OR = 1.17, 95% CI 0.94–1.46, *P*_H_ = 0.28; GG + GA versus AA: OR = 1.14, 95% CI 0.93–1.42, *P*_H_ = 0.42; and GG versus GA + AA: OR = 0.88, 95% CI 0.51–1.51, *P*_H_ = 0.73).

In the UC, the random effects model was used in the heterozygote model, dominant model, and recessive model, because of the presence of heterogeneity in the three models. A significant increased risk was identified in the homozygote model (GG versus AA: OR = 1.47, 95% CI 1.04–2.06, *P*_H_ = 0.1), while no obvious association was observed in other models (G versus A: OR = 1.11, 95% CI 0.96–1.29, *P*_H_ = 0.53; GA versus AA: OR = 0.82, 95% CI 0.41–1.63, *P*_H_< 0.001; GG + GA versus AA: OR = 0.97, 95% CI 0.69–1.36, *P*_H_ = 0.02; and GG versus GA + AA: OR = 1.19, 95% CI 0.43–3.30, *P*_H_< 0.001). In the CD, no significant statistical heterogeneity was identified in all models and hence the fixed effects model was used. No significant association existed (G versus A: OR = 1.02, 95% CI 0.81–1.28, *P*_H_ = 0.9; GG versus AA: OR = 1.29, 95% CI 0.67–2.5, *P*_H_ = 0.98; GA versus AA: OR = 0.94, 95% CI 0.71–1.24, *P*_H_ = 0.91; GG + GA versus AA: OR = 0.98, 95% CI 0.75–1.27, *P*_H_ = 0.99; and GG versus GA + AA: OR = 1.33, 95% CI 0.69–2.54, *P*_H_ = 0.99).

### 3.5. Sensitivity Analysis

**Figure 2:**
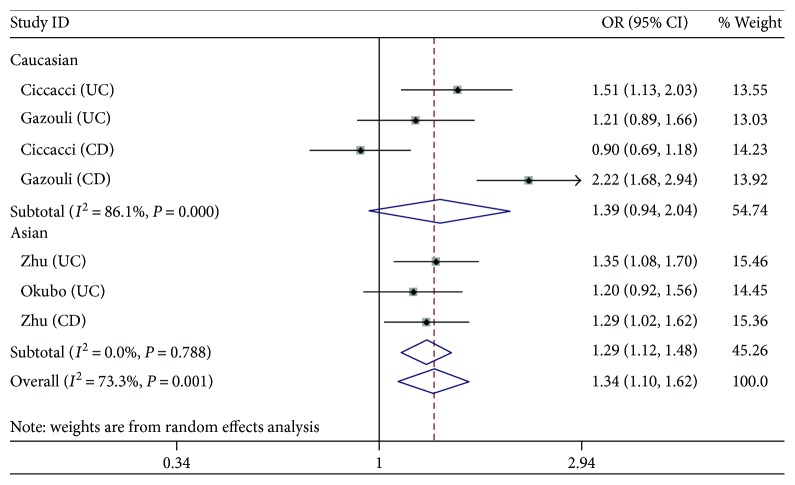
Forest plots of the OR with 95% CI for miRNA-146a rs2910164 (C versus G).

**Figure 3:**
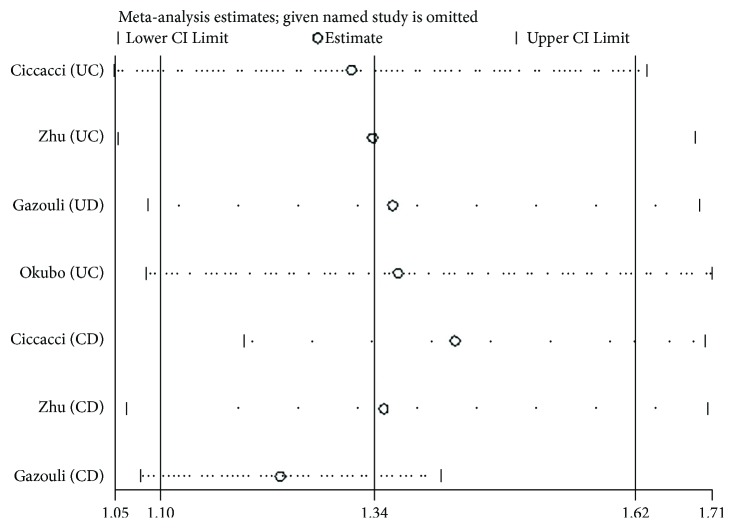
Sensitivity analyses by deleting one study every time.

**Figure 4:**
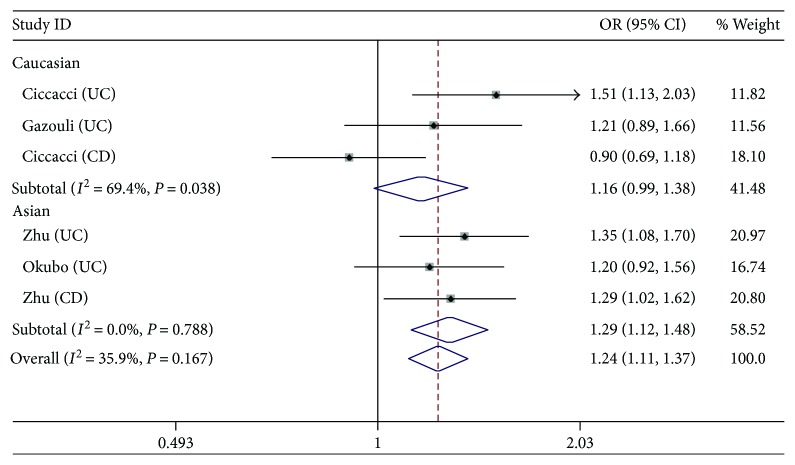
Forest plots of the OR with 95% CI for miRNA-146a rs2910164 after excluding the study from Okubo et al. (C versus G).

We performed the sensitivity analysis to test the influence of individual study on the pooled ORs by deleting each study every time (Figure 2). For *rs11614913* and *rs3746444*, the pooled estimate indicated that there was no significant difference. For *rs2910146*, the study from Okubo et al. [15] showed a significant effect on the pooled ORs. After excluding this study, the result of heterogeneity test was negative, and the pooled OR of allelic comparison C versus G turned to be 1.24 (95% CI: 1.11–1.37, *P* < 0.0001) (Figure 3). Nevertheless, the ORs of the homozygote model, heterozygote model, and dominant model remained significant, which indicated that the study about CD from Gazouli et al. might have a major influence on the pooled estimate (see Figure 4).

### 3.6. Publication Bias

**Figure 5:**
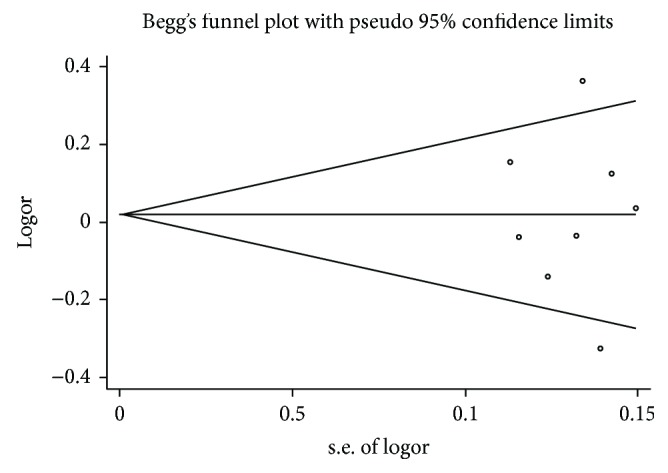
Begg's funnel plot for miRNA-196a2 rs11614913 (T>C) and IBD.

Publication bias was evaluated by Begg's funnel plot and Egger's test. No publication bias for the association between *miRNA-146a rs2910146* (G>C) and IBD susceptibility which was identified by Begg's funnel plot (*P* = 0.881, C versus G) and Egger's test (*P* = 0.793, C versus G). Begg's funnel plot (Figure 5) and Egger's test (*P* = 0.882, C versus T) between *miRNA-196a2 rs11614913* (T>C) and IBD susceptibility showed no publication bias. No test was assessed for the association between *miRNA-499 rs3746444* (A>G) and IBD susceptibility due to the limited number of selected studies.

## 4. Discussion

Accumulating emerged evidences had shown that there was specific relationship between miRNAs and autoimmunity diseases (ADs). A series of meta-analyses [19, 20], published from the year 2013 to the year 2015, summarized the potential association between ADs and miRNAs (*miRNA-146a rs2910146* and *miRNA-499 rs3746444*). However, no investigation was conducted to examine the relationship between IBDs and these three miRNAs. In the present meta-analysis, five eligible articles containing nine case-control studies were selected and 1761 cases and 1892 controls for the association between the three SNPs and IBD were analyzed. In our findings, there was no obvious association between *miRNA-196a2 rs11614913* and IBD, even in the subgroup analysis based on ethnicity or diseases. For *miRNA-146a rs2910146* and IBD, the associated susceptibility increased significantly. In allelic comparison, homozygote model, heterozygote model, and dominant model, similar results were obtained in Asian subgroups and UC subjects. In the Asian population, significant results were also observed in the recessive model. In addition, significant result existed in the reserved model of subgroup CD. As for *miRNA-499 rs3746444* and IBD, a positive relationship between them was identified in the homozygote model, and the same results were observed in subgroups Caucasian and UC.

It was reported that *miRNA-146a*, mapped to chromosome 5q33, was a nuclear factor-*κ*B- (NF-*κ*B-) dependent gene whose G/C polymorphism is located in the stem region (at position +60 relative to the first nucleotide of pre-miR-146a) opposite to the mature miR-146a precursor and could reduce the expression of tumor necrosis factor-receptor-associated factor-6 and interleukin-1 receptor- (ILR-) associated kinase-1, thus preventing excessive inflammation [21, 22]. Jazdzewski et al. [23] found that the *miRNA-146a rs2910146* polymorphism could cause additional generation of mature microRNAs from the passenger strands of the miRNA precursor. Regardless of the approaches the *miRNA-146a rs2910146* polymorphism influence the development of diseases, increasing studies had shown that rs2910146 (G>C) played an important role in the pathogenetic process of many diseases, for example, asthma [24] and rheumatoid arthritis [25] (which were both associated with immune disorders). Our results provided powerful evidences for the association between *miRNA-146a rs2910146* and susceptibility to IBD, for which research attention should be paid in future investigations. As one can expect, illuminating the inherent association between *miRNA-146a rs2910146* and IBD could help search new approaches for diagnosis and treatment [26, 27].

The strengths of our meta-analysis are as follows: (1) this is the first meta-analysis that is focused on the association between SNPs and IBD; (2) more updated studies were included in our research, compared to the previous meta-analyses about the association between SNPs and AD; (3) *miRNA-196a2 rs11614913* was investigated in our meta-analysis, which did not receive attention from previous research; and (4) the result remained significant after excluding heterogenic origins.

However, some limitations still exist in our studies. First of all, because of the shortage of original studies, the number of selected studies limited our further analysis. In addition, one of the included studies on *miRNA196* did not meet the HWE, though the pooled estimate remained insignificant. Moreover, obvious heterogeneity existed in *miRNA146a*, even in the subgroup Caucasian population. Although the sensitive analyses were conducted, the results need further supports from more well-designed studies with larger sample sizes. Finally, only three SNPs were included in our study, while there were more SNPs in other genes that could influence the susceptibility to IBD, and interactions among these SNPs and impacts of their network on diseases were not considered in our study.

In conclusion, our results suggested that *miRNA-146a* polymorphism was significantly associated with the susceptibility to IBD, especially in Asian and in UC. Nevertheless, in the future, more studies with larger samples as well as joint research on SNPs and the roles of SNPs in the pathogenesis of diseases and disorders are needed to further validate our results.

## Figures and Tables

**Table 1:** Characteristics of studies included in the meta-analysis.

Study	Year	Country	Ethnicity	Control			Diseases	Case			*P* for HWE	Quality score
miR196a2rs11614913 T>C				CC	TC	TT		CC	TC	TT		
Ranjha et al.	2017	India	Caucasian	305	81	55	UC	102	81	14	<0.0001	6
Ciccacci et al.	2017	Italy	Caucasian	101	115	23	IBD	171	172	38	0.234	8
							UC	94	91	21		
							CD	77	81	17		
Zhu et al.	2016	China	Asian	102	213	135	IBD	117	214	137	0.306	8
							UC	67	108	66		
							CD	50	106	71		
Gazouli et al.	2013	Greece	Caucasian	108	144	48	IBD	181	199	72	1	7
							UC	98	91	21		
							CD	83	108	51		
Okubo et al.	2011	Japan	Asian	75	206	122	UC	30	82	58	0.465	8
miRNA146a rs2910164 G>C	GG	CG	CC		GG	CG	CC					
Ciccacci et al.	2017	Italy	Caucasian	20	88	144	IBD	39	188	277	0.213	8
							UC	16	71	119		
							CD	23	117	158		
Zhu et al.	2016	China	Asian	97	202	151	IBD	62	225	181	0.059	7
							UC	32	113	96		
							CD	30	112	85		
Gazouli et al.	2013	Greece	Caucasian	200	90	10	IBD	231	191	30	0.974	7
							UC	126	78	6		
							CD	105	113	24		
Okubo et al.	2011	Japan	Asian	74	178	151	UC	28	67	75	0.095	8
miRNA499 rs3746444 A>G	AA	GA	GG		AA	GA	GG					
Ranjha et al.	2017	India	Caucasian	167	220	54	UC	97	35	65	0.154	8
Ciccacci et al.	2017	Italy	Caucasian	139	98	15	IBD	209	153	25	0.677	8
							UC	108	87	11		
							CD	101	66	14		
Zhu et al.	2016	China	Asian	339	105	6	IBD	357	105	6	0.504	8
							UC	185	54	2		
							CD	172	51	4		
Okubo et al.	2011	Japan	Asian	272	111	20	UC	102	62	6	0.055	7

**Table 2:** Polled ORs of SNPs and IBD.

	*n*	OR (95% CI) *P*	*P*_H_	OR (95% CI) *P*	*P*_H_	OR (95% CI) *P*	*P*_H_	OR (95% CI) *P*	*P*_H_	OR (95% CI) P	*P*_H_
miR196a2 C>T		T/C Allelic model		TT/CC Homozygote model		TC/CC Heterozygote model		TT + TC/CC Dominant model		TT/TC + CC Recessive model	
*Ethnicity*											
Overall		0.98 (0.85–1.13) *P* = 0.80	0.019	0.92 (0.76–1.11) *P* = 0.38	0.18	1.03 (0.74–1.43) *P* = 0.87	<0.001	1.04 (0.79–1.36) *P* = 0.80	<0.001	0.97 (0.83–1.14) *P* = 0.70	0.12
Caucasian		0.99 (0.78–1.25) *P* = 0.92	0.005	0.88 (0.60–1.27) *P* = 0.46	0.099	1.10 (0.66–1.83) *P* = 0.72	<0.001	1.10 (0.72–1.66) *P* = 0.67	<0.001	0.88 (0.59–1.30) *P* = 0.51	0.049
Asian		0.97 (0.90–1.07) *P* = 0.62	0.4	0.96 (0.73–1.25) *P* = 0.74	0.32	0.91 (0.71–1.16) *P* = 0.43	0.58	0.92 (0.74–1.16) *P* = 0.49	0.40	1.03 (0.83–1.14) *P* = 0.80	0.50
*Diseases*											
UC	5	0.94 (0.76–1.17) *P* = 0.59	0.007	0.79 (0.62–1.00) *P* = 0.06	0.23	1.07 (0.62–1.84) *P* = 0.82	<0.001	1 (0.64–1.53) *P* = 0.97	<0.001	0.87 (0.71–1.06) *P* = 0.16	0.11
CD	3	1.06 (0.92–1.22) *P* = 0.45	0.65	1.16 (0.86–1.56) *P* = 0.33	0.65	0.97 (0.77–1.22) *P* = 0.8	0.95	1.14 (0.92–1.41) *P* = 0.25	0.25	1.16 (0.9–1.48) *P* = 0.26	0.57
miRNA146a G>C		C/G Allelic model		CC/GG Homozygote model		CG/GG Heterozygote model		CC + CG/GG Dominant model		CC/CG + GG Recessive model	
*Ethnicity*											
Overall		1.34 (1.10–1.62) *P* = 0.003	0.001	1.56 (1.10–2.22) *P* = 0.001	0.037	1.50 (1.17–1.93) *P* = 0.04	0.081	1.54 (1.17–2.02) *P* = 0.02	0.022	1.19 (0.95–1.49) *P* = 0.13	0.062
Caucasian		1.39 (0.94–2.04) *P* = 0.097	<0.001	1.45 (0.68–3.08) *P* = 0.34	0.009	1.50 (1.00–2.24) *P* = 0.001	0.051	1.46 (0.90–2.34) *P* = 0.01	0.008	1.18 (0.72–1.92) *P* = 0.51	0.019
Asian		1.29 (1.12–1.48) *P* < 0.001	0.79	1.69 (1.27–2.24) *P* < 0.001	0.52	1.49 (1.31–1.87) *P* = 0.005	0.198	1.61 (1.24–2.08) *P* = 0.25	0.25	1.27 (1.04–1.54) *P* = 0.02	0.886
*Diseases*											
UC	4	1.32 (1.15–1.50) *P* < 0.001	0.64	1.43 (1.06–1.93) *P* = 0.02	0.39	1.32 (1.04–1.67) *P* = 0.02	0.42	1.38 (1.10–1.73) *P* = 0.34	0.34	1.20 (0.99–1.47) *P* = 0.07	0.65
CD	3	1.37 (0.85–2.22) *P* = 0.021	0	1.94 (0.89–4.24) *P* = 0.10	0.009	1.94 (1.49–2.52) *P* < 0.001	0.15	1.79 (1.10–2.02) *P* = 0.03	0.034	1.33 (0.76–2.33) *P* = 0.32	0.006
miRNA499 A>G		G/A		GG/AA		GA/AA		GG + GA/AA		GG/GA + AA	
*Ethnicity*											
Overall		1.08 (0.96–1.22) *P* = 0.20	0.75	1.43 (1.06–1.93) *P* = 0.02	0.27	0.86 (0.56–1.33) *P* = 0.50	<0.001	0.97 (0.78–1.20) *P* = 0.77	0.06	1.28 (0.64–2.53) *P* = 0.49	0.001
Caucasian		1.08 (0.92–1.28) *P* = 0.37	0.38	1.72 (1.19–1.49) *P* = 0.004	0.24	0.62 (0.29–1.35) *P* = 0.23	<0.001	0.81 (0.66–1.00) *P* = 0.051	0.17	1.74 (0.68–4.49) *P* = 0.25	0.016
Asian		1.09 (0.91–1.30) *P* = 0.37	0.70	0.94 (0.54–1.64) *P* = 0.84	0.83	1.17 (0.94–1.46) *P* = 0.16	0.28	1.14 (0.93–1.42) *P* = 0.21	0.42	0.88 (0.51–1.51) *P* = 0.63	0.73
*Diseases*											
UC	4	1.11 (0.96–1.29) *P* = 0.15	0.53	1.47 (1.04–2.06) *P* = 0.03	0.1	0.82 (0.41–1.63) *P* = 0.57	<0.001	0.97 (0.69–1.36) *P* = 0.15	0.02	1.19 (0.43–3.30) *P* = 0.73	0
CD	2	1.02 (0.81–1.28) *P* = 0.89	0.9	1.29 (0.67–2.5) *P* = 0.45	0.98	0.94 (0.71–1.24) *P* = 0.68	0.91	0.98 (0.75–1.27) *P* = 0.86	0.99	1.33 (0.69–2.54) *P* = 0.40	0.99

*P*: *P* value with significance. *P* < 0.05: significant statistical difference among different data. *P*_H_: *P* value with heterogeneity. *P*_H_ < 0.1: significant statistical heterogeneity among various data. The random effects model should be applied; otherwise, instead, the fixed effects model should be used.
